# Heart–Lungs interactions: the basics and clinical implications

**DOI:** 10.1186/s13613-024-01356-5

**Published:** 2024-08-12

**Authors:** Mathieu Jozwiak, Jean-Louis Teboul

**Affiliations:** 1 Service de Médecine Intensive Réanimation, CHU de Nice Hôpital Archet 1, 151 Route Saint Antoine de Ginestière, 06200 Nice, France; 2 UR2CA, Unité de Recherche Clinique Côte d’Azur, Université Côte d’Azur, 06200 Nice, France; 3 Faculté de Médecine Paris-Saclay, Université Paris-Saclay, 94270 Le Kremlin-Bicêtre, France

**Keywords:** Cardiac loading conditions, Intrathoracic pressure, Fluid responsiveness, Transpulmonary pressure

## Abstract

Heart–lungs interactions are related to the interplay between the cardiovascular and the respiratory system. They result from the respiratory-induced changes in intrathoracic pressure, which are transmitted to the cardiac cavities and to the changes in alveolar pressure, which may impact the lung microvessels. In spontaneously breathing patients, consequences of heart–lungs interactions are during inspiration an increase in right ventricular preload and afterload, a decrease in left ventricular preload and an increase in left ventricular afterload. In mechanically ventilated patients, consequences of heart–lungs interactions are during mechanical insufflation a decrease in right ventricular preload, an increase in right ventricular afterload, an increase in left ventricular preload and a decrease in left ventricular afterload. Physiologically and during normal breathing, heart–lungs interactions do not lead to significant hemodynamic consequences. Nevertheless, in some clinical settings such as acute exacerbation of chronic obstructive pulmonary disease, acute left heart failure or acute respiratory distress syndrome, heart–lungs interactions may lead to significant hemodynamic consequences. These are linked to complex pathophysiological mechanisms, including a marked inspiratory negativity of intrathoracic pressure, a marked inspiratory increase in transpulmonary pressure and an increase in intra-abdominal pressure. The most recent application of heart–lungs interactions is the prediction of fluid responsiveness in mechanically ventilated patients. The first test to be developed using heart–lungs interactions was the respiratory variation of pulse pressure. Subsequently, many other dynamic fluid responsiveness tests using heart–lungs interactions have been developed, such as the respiratory variations of pulse contour-based stroke volume or the respiratory variations of the inferior or superior vena cava diameters. All these tests share the same limitations, the most frequent being low tidal volume ventilation, persistent spontaneous breathing activity and cardiac arrhythmia. Nevertheless, when their main limitations are properly addressed, all these tests can help intensivists in the decision-making process regarding fluid administration and fluid removal in critically ill patients.

## Background

**Fig. 1:**
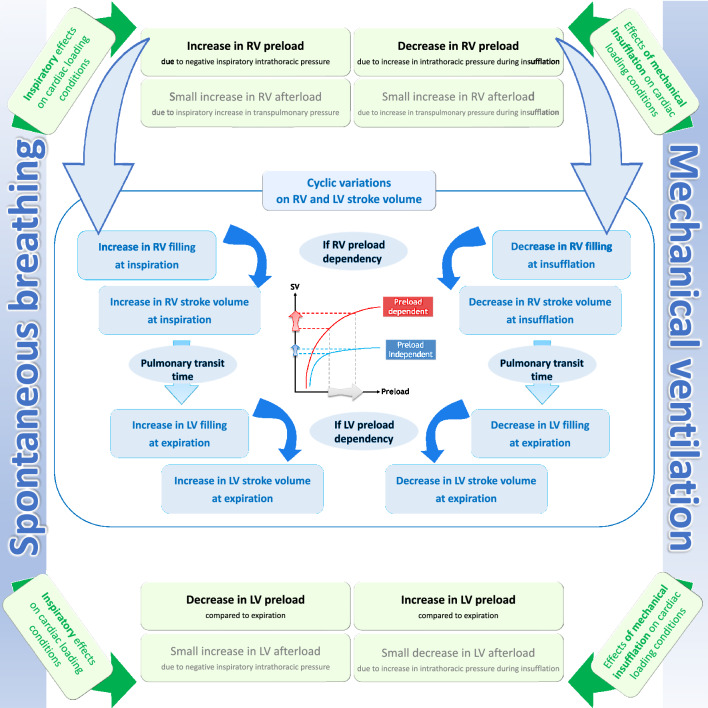
Summary of heart–lungs interaction in spontaneously breathing patients and in mechanically ventilated patients. In physiological conditions (spontaneously breathing), inspiratory increase in right ventricular (RV) preload and decrease in left ventricular (LV) preload are the two predominant global effects of ventilation on cardiac loading conditions. In patients with healthy lungs and heart (mechanical ventilation), decrease in RV preload and increase in LV preload during insufflation are the two predominant global effects of ventilation on cardiac loading conditions

The hemodynamic consequences of heart–lungs interactions result from the fact that in the confined space of the thorax, the cardiovascular system on the one hand and the respiratory system on the other hand are subject to different pressure regimes. Physiologically and during normal breathing, heart–lungs interactions do not lead to significant hemodynamic consequences. This is not the case during acute exacerbation of asthma or chronic obstructive pulmonary disease, acute left heart failure and during weaning from mechanical ventilation. In the first part of this review, heart–lungs interactions in spontaneously breathing and then in mechanically ventilated patients will be described (Fig. 1). In the second part of this review, heart–lungs interactions and their potential harmful or beneficial hemodynamic impact in different clinical settings as well as their potential clinical implications will be discussed.

## Heart–lungs interactions in spontaneously breathing patients

A simple way to describe heart–lungs interactions is to consider the interactions between two pumps: the smaller (circulatory pump) being contained within the larger (respiratory pump). While the respiratory pump acts as a suction pump, developing a negative pressure to allow air entry into the airways and blood into cardiac cavities, the circulatory pump acts as a pressure pump, developing a positive pressure to eject blood towards the arterial tree. As the circulatory pump is contained within the thorax, the circulatory pump is affected by the pressures generated by the respiratory pump [1].

### Initiating phenomena

#### Intrathoracic pressure negativity

Spontaneous inspiration is responsible for a negative intrathoracic pressure. The difference between the intrathoracic pressure and the alveolar pressure must increase during inspiration so that the latter becomes lower than the atmospheric pressure and allows air entering the airways.

#### Increase in intra-abdominal pressure

The negative intrathoracic pressure is essentially driven by the diaphragm which lowers at inspiration and increases the intra-abdominal pressure: the thorax and abdomen have opposite pressure regimes at inspiration [2].

### Cardiac consequences

#### Inspiratory increase in right ventricular preload

The systemic venous return to the right atrium is driven by the pressure gradient between the upstream capacitive venous system where the mean systemic pressure prevails and the downstream right atrium. Thus, the systemic venous return is closely linked to the right atrial pressure: the more the right atrial pressure decreases, the more the venous return increases [3]. At inspiration, the negativity of the intrathoracic pressure is transmitted to the right atrium, thus increasing the pressure gradient between the extrathoracic venous territory and the right atrium. Simultaneously, the increase in intra-abdominal pressure due to the descent of the diaphragm, contributes to the increase in this gradient since it increases the mean systemic pressure and drives venous blood into the thorax [3].

#### Inspiratory increase in right ventricular afterload

**Fig. 2:**
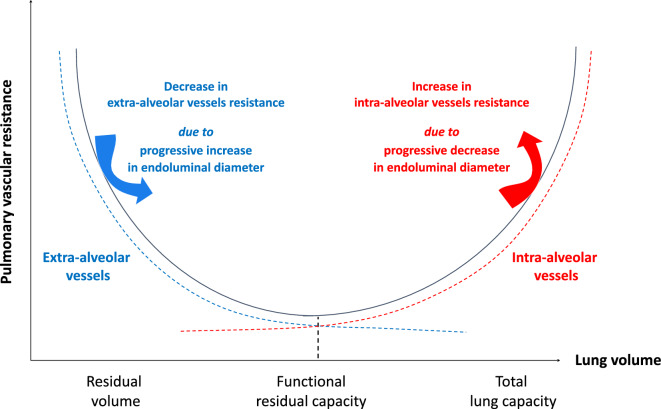
Relationship between pulmonary vascular resistance and lung volume. The dotted blue line represents the pulmonary vascular resistance of the extra-alveolar vessels. The dotted red line represents the pulmonary vascular resistance of the intra-alveolar vessels

From a serial component viewpoint, the pulmonary circulation may be divided in extra-alveolar vessels and intra-alveolar vessels [4]. Lung volume expansion during inspiration compresses lumens of intra-alveolar vessels resulting in an exponential increase in intra-alveolar vessels resistance. By contrast, increase in lung volume induces an exponential decrease in extra-alveolar vessels resistance. Indeed, as lung volume increases the radial interstitial forces increase, resulting in widening of extra-alveolar vessels diameters. Thus, the resulting total pulmonary vascular resistance describes a U shape with a nadir corresponding to a lung volume equal to the functional residual capacity (FRC) (Fig. 2) [4–6].

**Fig. 3:**
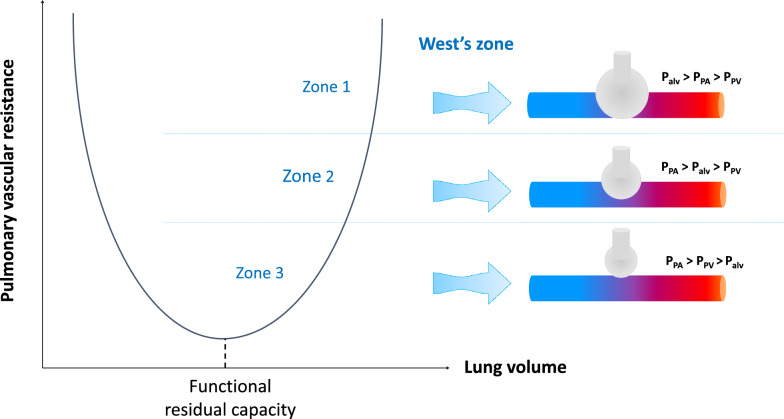
Concept of the pulmonary West’s zone illustrating the distribution of the pulmonary circulation along a gravitational gradient of the vascular-alveolar pressure difference. *P*_*alv*_ alveolar pressure, *P*_*PV*_ pulmonary venous pressure, *P*_*PA*_ pulmonary artery pressure

From a parallel component viewpoint, the pulmonary circulation is distributed along a gravitational gradient of the vascular-alveolar pressure difference (Fig. 3) [5]. Accordingly, by decreasing intrathoracic pressure more than alveolar pressure, spontaneous inspiration may cause a larger proportion of the pulmonary circulation to behave as West’s zone 2, especially when the pulmonary venous pressure is low. Consequently, pulmonary vascular resistance and right ventricular afterload may increase, at least during inspiration. It is noteworthy that in case of normal breathing conditions (no deep inspiratory efforts, normal compliance of the respiratory system) the difference between the inspiratory decrease in intrathoracic pressure and the inspiratory decrease in alveolar pressure is small so that the inspiratory increase in pulmonary vascular resistance will be of minor degree.

#### Inspiratory decrease in left ventricular preload

First, the inspiratory increases in right ventricular preload and afterload may induce an increase in right ventricular volume during inspiration. This will result in an inspiratory increase in right ventricular stroke volume if the right ventricle is preload-dependent. This increase will be transmitted to the left ventricle during the following expiration because of the long pulmonary transit time (several seconds). This serial ventricular interdependence will thus contribute to lower left ventricular filling and preload during inspiration than during expiration.

Second, the increase in right ventricular volume during inspiration could result in a discrete decrease in left ventricular filling [7] due to the mechanism of parallel ventricular interdependence [8]. The latter is related to the fact that the heart is contained within the pericardium, a non-extensive envelope with high elastance, resulting in a constant sum of the volumes of the right and left ventricles. Finally, the small inspiratory increase in transpulmonary pressure could result in a shift of blood from the pulmonary venous circulation to the left atrium. Nevertheless, this mechanism is probably of minor importance since studies in normal subjects showed a decrease in left ventricular preload during inspiration [7, 9, 10]. It is likely that the parallel and more importantly the serial ventricular interdependence phenomena are responsible for the decrease in left ventricular preload during inspiration, which eventually results in a decrease in left ventricular stroke volume (if the left ventricle is preload-dependent) [9, 10] and thus in arterial pulse pressure [9] during inspiration.

#### Inspiratory increase in left ventricular afterload

The left ventricular afterload can be thought as the effort required by the left ventricle to eject blood up to the level of pressure of the extrathoracic vessels (atmospheric pressure for the vessels of the neck and upper limbs, intra-abdominal pressure for the abdominal aorta). At inspiration, the negative intrathoracic pressure places the left ventricle at a lower level and makes its ejecting effort greater, thus increasing the left ventricular afterload [11, 12]. The hemodynamic consequences of an increase in left ventricular afterload are negligible in patients with normal left ventricular function due to the physiological relative cardiac "afterload-independence". In this regard, studies in normal subjects showed a small reduction (and not an augmentation) of left ventricular end-diastolic volume [7, 9, 10] due to the above-mentioned mechanisms.

In summary, the cardiac consequences of spontaneous inspiration are an increase in right ventricular preload and afterload, a decrease in left ventricular preload and an increase in left ventricular afterload. However, during quiet spontaneous breathing in normal humans, these consequences are of limited degree resulting *in fine* in a small decrease in left ventricular stroke volume, arterial pulse and systolic pressures at inspiration. However, in some pathological conditions, the cardiac consequences can be of major importance (see below).

## Heart–lungs interactions in mechanically ventilated patients

### Initiating phenomena

During mechanical ventilation, the alveolar and intrathoracic pressures are positive during the entire respiratory cycle with a minimum at end-expiration. Heart–lungs interactions under mechanical ventilation are related to the impairment of right ventricular filling and ejection due to the increase in these pressures.

### Cardiac consequences

#### Decrease in right ventricular preload during insufflation

During mechanical insufflation, the increase in intrathoracic pressure is transmitted to the right atrium. This should reduce the pressure gradient between the venous system and the right atrium and thus should decrease the systemic venous return [13]. However, other mechanisms can be involved. During mechanical insufflation, the intra-abdominal pressure should also increase, which in turn should increase the mean systemic pressure by facilitating blood redistribution from the unstressed to the stressed blood volume. This effect can be limited if the unstressed blood volume is low (e.g. in case of volume depletion). Baroreceptor-related sympathetic stimulation could also increase mean systemic pressure during mechanical insufflation. These two latter mechanisms can limit the decrease in the venous return pressure gradient so that during normal tidal insufflation, the decrease in venous return is small.

#### Increase in right ventricular afterload during insufflation

As for spontaneous breathing, two different mechanisms are involved in the inspiratory increase in right ventricular afterload. The first mechanism is related to the U-shape relationship between pulmonary vascular resistance and lung volume [6]. In normal lung conditions, the end-expiratory lung volume equals the FRC so that mechanical insufflation increases the pulmonary vascular resistance by an amount depending on tidal volume in an exponential way. The second mechanism is related to the more pronounced increase in alveolar pressure than intrathoracic pressure during insufflation, which may potentially result in a transfer of West’s zone 3 to West’s zone 2. This mechanism may occur when the alveolar pressure becomes higher than the pulmonary venous pressure during insufflation. The main conditions of this occurrence are the presence of a low pulmonary venous pressure (e.g. in case of low central blood volume) and a marked insufflation-related increase in transpulmonary pressure (alveolar pressure minus intrathoracic pressure) due to high tidal volume ventilation or to reduced lung compliance, which reduces the airway pressure transmission [14, 15]. So, the lower the lung compliance (or the lower the compliance of the respiratory system), the lower the transmission of the airway pressure and the higher the transpulmonary pressure. For all these reasons, mechanical insufflation should increase pulmonary vascular resistance and right ventricular afterload [16]. However, in patients with normal compliance of the respiratory system, this effect should be limited if low tidal volume ventilation is used, what is usually the case in critically ill patients who receive mechanical ventilation.

#### Increase in left ventricular preload during insufflation

First, the increase in transpulmonary pressure during insufflation may induce a shift of blood from the pulmonary venous circulation to the left atrium, thus increasing the filling of the left ventricle at the same time [17]. Second, due to the serial ventricular interdependence, the decrease in systemic venous return combined with the impeded right ventricular ejection during insufflation result in a decrease in the left ventricular filling and preload during the following expiration because of the long pulmonary transit time. In accordance with these mechanisms, an increase in echocardiographic indexes of left ventricular preload during insufflation was demonstrated [17]. It is noteworthy that if the right ventricular ejection is markedly impeded during insufflation, right ventricular overload might occur and result in a leftward septal shift. This septal shift during insufflation could reduce the distensibility of the left ventricle and impedes its filling through the parallel ventricular interdependence [8, 18]. Nevertheless, even if the right ventricular stroke volume decreases during insufflation [17, 19] due to the combined effects of decrease in right ventricular preload and increase in right ventricular afterload, unchanged and not enlarged right ventricular end-diastolic volume during insufflation was reported [17, 19] making the occurrence of a pronounced leftward septal shift during insufflation unlikely.

#### Decrease in left ventricular afterload during insufflation

Mechanical ventilation decreases the left ventricular afterload during inspiration and therefore should facilitate the left ventricular ejection. This transient and “paradoxical” phenomenon may be explained by a brief synergy between the respiratory and the circulatory pumps, both of which develop positive pressure at the same time. The increase in intrathoracic pressure is transmitted to the left ventricle and the intrathoracic part of the aorta, resulting in a decrease in the transmural aortic pressure. Thus, during insufflation, the positive intrathoracic pressure places the left ventricle at a higher level and makes its ejection effort lower, thus decreasing the left ventricular afterload [11, 12]. This effect could have a positive impact on left ventricular stroke volume in case of left ventricular afterload-dependence, a phenomenon sometimes observed in patients with left ventricular dysfunction.

In summary, right and left ventricular loading conditions vary over the respiratory cycle. This leads to a higher left ventricular stroke volume and hence of arterial pulse and systolic pressures during insufflation than during expiration. To distinguish between a true increase in systolic arterial pressure during insufflation compared to apneic conditions and a true decrease of systolic arterial pressure during expiration compared to apneic conditions, it was proposed to observe the change in the arterial pressure signal during a brief interruption of the ventilator at end-expiration [20]. The delta Up (Δup) component—the difference between the maximal systolic arterial pressure and the apneic systolic arterial pressure—should reflect the true increase in left ventricular stroke volume during insufflation due to either the blood shift from the pulmonary capillaries to the left atrium [17] and/or the left ventricular afterload decrease (see above). The delta Down (Δdown) component—the difference between the apneic systolic arterial pressure and the minimal systolic arterial pressure—should reflect the true decrease in left ventricular stroke volume during expiration due to the time-delayed (long pulmonary transit time) decrease in right ventricular stroke volume during insufflation. The delta Up component could be predominant in case of congestive heart failure while the delta Down component could be predominant in case of hypovolemia [20]. Finally, as we detail below, the magnitude of the variation of left ventricular stroke volume and thus of arterial pulse pressure during mechanical ventilation has been proposed to identify fluid responsiveness.

## Heart–lungs interactions in clinical settings and clinical implications

In several clinical settings, heart–lungs interactions may lead to significant hemodynamic consequences because of specific pathophysiological mechanisms, may explain the interest of some therapeutics and may have clinical implications. Heart–lungs interactions in these different clinical settings will be briefly summarized below.

### Acute exacerbation of chronic obstructive pulmonary disease

**Fig. 4:**
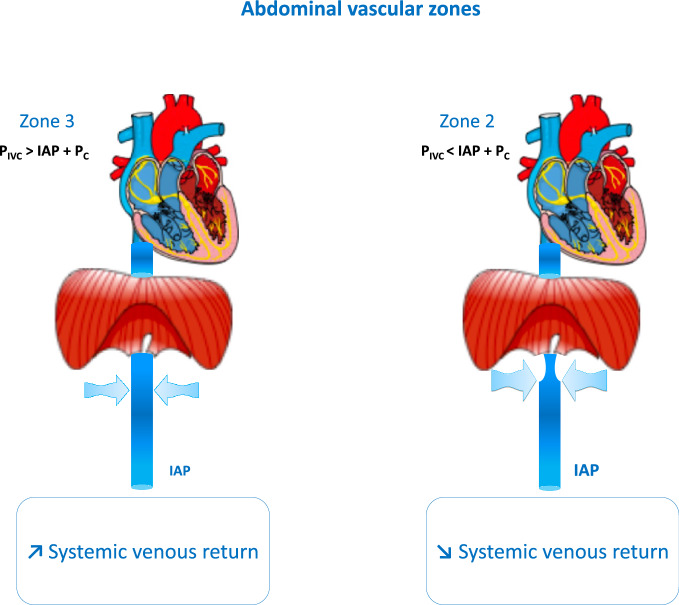
Concept of abdominal vascular zone conditions illustrating the effects of intra-abdominal pressure on systemic venous return. *IAP* intra-abdominal pressure, *P*_*IVC*_ intramural pressure of inferior vena cava at the level of the diaphragm, *P*_*C*_ critical closing transmural pressure

Dynamic hyperinflation is one of the characteristics of acute exacerbation of characteristics of chronic obstructive pulmonary disease (COPD). Such a phenomenon is favored by (i) increased airway resistance related to bronchoconstriction, mucosal oedema and excessive sputum, (ii) decrease elastic recoil pressure, (iii) tachypnea, which reduces the time devoted to expiration, and (iv) mainly expiratory airflow limitation. This results in an end-expiratory lung volume higher than the relaxation lung volume and therefore in a positive static end-expiratory elastic recoil pressure called intrinsic positive end-expiratory pressure (PEEP). This leads to accentuated inspiratory negativity of intrathoracic pressure and increased work of breathing [21]. These events should increase cardiac output to meet increased oxygen demand. Increased sympathetic activity leading to tachycardia participates in this response. The marked negativity of the intrathoracic pressure also contributes to increasing systemic venous return and cardiac output since it decreases the right atrial pressure. At the same time the increase in intra-abdominal pressure increases the mean systemic pressure so that the venous return pressure gradient should increase. However, in case of a very marked inspiratory negativity of intrathoracic pressure, the intra-abdominal pressure may become so positive in relation to the right atrial pressure that it leads to collapse of the inferior vena cava in its subdiaphragmatic segment, which interrupts the inspiratory increase in systemic venous return (Fig. 4) [22, 23]. This phenomenon may occur mostly when patients are hypovolemic [23]. In addition, due to the marked negativity of the intrathoracic pressure compared to the alveolar pressure, the right ventricular afterload should increase more during acute exacerbation than during quiet breathing conditions in patients with COPD. Moreover, worsening of hypoxemia during acute exacerbation of COPD may aggravate the pulmonary hypertension and hence induce a more marked increase in the right ventricular afterload through the hypoxic pulmonary vasoconstriction mechanism. The role of hypercapnia on pulmonary vascular resistance is less clear as it was reported to have a pulmonary vasodilatory or a pulmonary vasoconstricting effect depending on some experimental conditions [24]. However, it is likely that during hypoxemia, hypercapnia should further increase pulmonary vasoconstriction [24]. In patients with prior right ventricular dysfunction, as it is sometimes the case in COPD, an additional increase in right ventricular afterload may further worsen the right ventricular dysfunction [25] and may potentially lead to decreased stroke volume.

In summary, systemic venous return should normally increase during acute exacerbation of COPD, in particular during inspiration. However, marked inspiratory efforts with exaggerated drops in intrathoracic pressure may result in reduction in venous return due to flow limitation of the inferior vena cava and increased abdominal pressure, especially when the intravascular volume is low. On the other hand, in some conditions, right ventricular afterload may markedly increase during acute exacerbation of COPD. In case of previously dilated right ventricle, left ventricular filling can be limited through biventricular interdependence resulting in decreased stroke volume and increased left ventricular filling pressure. For patients with history of COPD and chronic left ventricular dysfunction presenting with acute respiratory failure, it is sometimes difficult to distinguish clinically between acute exacerbation of COPD and cardiogenic pulmonary oedema since the former could favor the latter due to heart–lungs interactions. This emphasizes the need for individualized assessment at least using echocardiography before administering any treatment. For example, deliberate administration of diuretics in this situation could be risky if the episode of acute respiratory failure is only related to acute exacerbation of COPD.

### Acute left heart failure

In spontaneously breathing patients with acute heart failure, the same initiating phenomena than those described above for patients with acute exacerbation of chronic obstructive pulmonary disease may participate to the hemodynamic consequences of heart–lungs interactions. In this clinical setting, the accentuated inspiratory negativity of intrathoracic pressure [26] is related to reduced lung compliance and increased airway resistance [26]. The reduced lung compliance is a consequence of interstitial and/or alveolar oedema. The increase in airway resistance may be related to several mechanisms [27, 28]: (i) a bronchial wall thickening because of bronchial oedema formation and/or increased vascular volume, (ii) a reflex bronchoconstriction of vagal origin, stimulated by increased pulmonary vascular pressures and/or interstitial or peribronchial oedema and/or (iii) a bronchial hyperreactivity. The marked decrease in intrathoracic pressure during inspiration along with the increase in intra-abdominal pressure should markedly increase the left ventricular afterload with potential decrease in the left ventricular stroke volume since the left ventricle is dependent on its afterload when it is failing. In addition, the hypoxic pulmonary vasoconstriction may accentuate the increase in the right ventricular afterload.

Heart–lungs interactions also explain the beneficial effects of positive pressure ventilation on the cardiovascular system in patients with acute left ventricular heart failure [29] in contrast to what occurs in patients with healthy heart and justify the use of non-invasive mechanical ventilation for the treatment of severe pulmonary oedema [30].

First, positive pressure ventilation, when PEEP is added, reduces the venous return pressure gradient by increasing the right atrial pressure. This could lead to a decrease in the right ventricular preload and central blood volume. The PEEP-induced decrease in cardiac preload and central blood volume may be particularly beneficial in patients with heart failure with preserved ejection fraction, as non-failing left ventricle is more preload-dependent than afterload-dependent.

Second, the use of positive pressure ventilation, when PEEP is added, may also improve left ventricular function through a decrease in left ventricular afterload [29, 31–34]. This effect is secondary to both the attenuation and suppression of the inspiratory negativity of intrathoracic pressure [26, 31]. The PEEP-induced decrease in left ventricular afterload may be particularly beneficial in patients with heart failure with reduced ejection fraction, as failing left ventricle is more afterload-dependent than preload-dependent. Thus, while positive pressure ventilation decreases stroke volume in patients with normal cardiac function, it increases it in patients with left ventricular dysfunction [33, 34].

A third beneficial effect of positive pressure ventilation with PEEP both in patients with acute heart failure with preserved or reduced ejection fraction is the alleviation of possible myocardial ischemia by restoring the balance between myocardial oxygen supply and demand. The increase in myocardial oxygen supply results from the restoration of arterial oxygenation and the improvement of coronary perfusion by reducing left ventricular end-diastolic pressure, the downstream pressure of coronary perfusion. The decrease in myocardial oxygen demand results from the decrease in work of the respiratory muscles [26] which, in respiratory failure, have a considerable oxygen consumption [35], thus reducing blood flow to the respiratory muscles and redistributing it to other organs [36].

All these theoretical advantages of non-invasive ventilation in patients with acute left heart failure have been demonstrated in many clinical trials showing clinical benefits in terms of clinical and/or oxygenation improvement [30, 37–40].

### Cardiac dysfunction induced by weaning from mechanical ventilation

Echocardiographic studies have shown that left ventricular diastolic dysfunction and increased left ventricular filling pressure are common during weaning failure [41, 42]. In a high percentage of cases of weaning failure, a cardiogenic pulmonary oedema may occur [43]. The risk factors for weaning-induced pulmonary oedema (WIPO) are COPD, cardiopathy (dilated and/or hypertrophic and/or hypokinetic cardiopathy and/or significant valvular disease) and obesity [43].

The WIPO is mainly induced by the shift from a positive to a negative pressure ventilation after disconnecting the ventilator [44–46]. The inspiratory negativity of intrathoracic pressure may be accentuated by the resistance of the chest tube [47]. This results in the heart–lungs interactions described above in spontaneously breathing patients, leading to unfavorable loading cardiac conditions (increase in right ventricular preload and afterload and increase in left ventricular afterload) and eventually to WIPO [46]. In patients with chronic right ventricular dysfunction, right ventricular enlargement during weaning can play a role in the development of WIPO through a biventricular interdependence mechanism. In patients with chronic left ventricular dysfunction, increase in left ventricular afterload due to accentuated negativity of intrathoracic pressure, increased intra-abdominal pressure and sympathetic-related arterial hypertension should also play an important role in the occurrence of WIPO. In any case, a positive fluid balance also contributes to WIPO [48].

Some studies also suggested a potential role of myocardial ischemia in the development of WIPO. Myocardial ischemia would be related to the increase in cardiac work (secondary to the increased work of breathing), the increase in left ventricular afterload and to the decrease in coronary perfusion [49, 50]. Nevertheless, recent findings suggest that myocardial ischemia plays no major role in the pathophysiology of WIPO [43, 48, 51].

Finally, it is well-established that left ventricular diastolic dysfunction is also involved in the pathophysiology of WIPO [41, 42, 48], while the potential role of left ventricular systolic dysfunction remains unclear [42] and is the subject of ongoing study (SystoWean study, NCT05226247).Since WIPO can be secondary to different mechanisms, it is important not only to diagnose it (for example using changes in hemoconcentration parameters during a weaning trial) but to identify what are the main underlying mechanism(s) in order to apply the most appropriate treatment [46].

### Acute respiratory distress syndrome

As detailed above, the main heart–lungs interactions include the effects of intrathoracic pressure on right ventricular preload and left ventricular afterload and the effects of transpulmonary pressure on right ventricular afterload, making lung compliance (or compliance of the respiratory system) one of the main important variables in heart–lungs interactions. Due to both the decrease in airway pressure transmission secondary to reduced lung compliance [14, 15] and the low tidal volume ventilation strategies [52], the changes in intrathoracic pressure induced by mechanical ventilation are expected to be too small to markedly alter hemodynamics over the ventilatory cycle in patients with acute respiratory distress syndrome (ARDS). Although the respiratory changes in transpulmonary pressure should be less negligible than the changes in intrathoracic pressure due to low airway pressure transmission [14, 15], the use of low tidal volume ventilation strategies should attenuate the changes in right ventricular afterload during the ventilatory cycle [19].

More significant are the hemodynamic effects of PEEP in patients with ARDS. The expected benefits of PEEP application are the reduction of non-aerated lung and improvement of arterial oxygenation [52]. The risks of PEEP application are lung overdistension, atelectrauma and hemodynamic instability due to decrease in cardiac output. The appropriate level of PEEP should be individualized, although there is no current strong recommendation on how to titrate PEEP [52]. The impact of PEEP on hemodynamics may involve its effect on the right ventricular afterload through the increase of transpulmonary pressure and/or its effect on the right ventricular preload through the increase in intrathoracic pressure. In patients with ARDS, the right ventricular afterload is already increased due to several mechanisms that increase the pulmonary vascular resistance. These mechanisms include hypoxic pulmonary vasoconstriction, mediators-related pulmonary vasoconstriction, microthrombi formation in pulmonary vessels, and pulmonary vascular remodeling. Even when lung protective ventilation is applied, acute cor pulmonale is observed in 20–25% of cases [53], probably due to the above-mentioned mechanisms.

In this context, the role of PEEP on the right ventricular afterload depends on its impact on lung mechanics. If PEEP only recruits closed alveoli units, the end-expiratory lung volume would increase toward the FRC so that the pulmonary vascular resistance would decrease. By contrast, if PEEP creates overdistension of lung units, it will increase the resistance of intra-alveolar vessels of these units and therefore will increase the pulmonary vascular resistance. The lungs of patients with ARDS include both closed alveoli units, which could be re-opened, and normal alveoli units, which could be overdistended. Thus, the impact of PEEP on pulmonary vascular resistance would depend on the recruitment/overdistension ratio for a given patient and at a given time of the disease, as it was illustrated in a recent clinical study [54]. This maybe explains why different responses of the right ventricular afterload to PEEP were reported in ARDS patients [55–59]. It is noteworthy that if PEEP improves gas exchange, it should decrease hypoxic vasoconstriction and thus decrease the pulmonary vascular resistance.

In addition, PEEP could exert effects on the systemic venous return determinants. By increasing the intrathoracic pressure, the right atrial pressure, which is the downstream pressure to systemic venous return should increase, although the reduced airway pressure transmission during ARDS should attenuate this effect [14, 15]. On the other hand, the mean systemic pressure (i.e. the upstream pressure to systemic venous return) should also increase and therefore limit the effects of the increase in intrathoracic pressure on venous return due to combined effects of the PEEP-induced increased intra-abdominal pressure [60] and to other adaptive mechanisms [61] including sympathetic-mediated mechanisms. In particular, the activation of the renin–angiotensin–aldosterone system during positive pressure ventilation may increase the mean systemic pressure by inducing a venoconstriction of the splanchnic vasculature which in turn results in a shift of blood into the systemic circulation [62]. Nevertheless, both the PEEP-induced increased intra-abdominal pressure and the other adaptive mechanisms may also increase venous resistance in some extent [61, 63]. If during ARDS, it seems thus unlikely that PEEP would markedly reduce cardiac output through a primary impact on systemic venous return, such a mechanism cannot be excluded in patients who receive heavy sedation able to blunt the adaptive responses to PEEP.

Previous clinical data have suggested that the decrease in right ventricular preload secondary to decreased systemic venous return may play an important role in the PEEP-induced decrease in cardiac output [55–57, 64, 65]. Others have suggested a predominant role of the increased right ventricular afterload [59, 66]. Many of the following factors could explain such divergent results: the capacity of PEEP to induce lung recruitment vs. overdistension, its capacity of improving arterial oxygenation, the amount of tidal volume, the degree of airway pressure transmission, the level of sedation, the degree of right ventricular preload-dependence and afterload-dependence, and the degree of left ventricular preload-dependence and afterload-dependence. Finally, the volume status also plays a key role. In case of decreased central blood volume (i.e. due to volume depletion), a larger proportion of the lungs are under West’s zone 2 conditions, so that the pulmonary vascular resistance and the right ventricular afterload should increase. The importance of this phenomenon was illustrated in a study including patients with ARDS [66]. In this study, pulmonary thermodilution and echocardiography parameters showed first an impairment of right ventricular function when PEEP was increased from 5 cmH_2_O to the level judged appropriate by the attending physician (on average 13 cmH_2_O) and then a return of the right ventricular function to the pre-PEEP condition during passive leg raising, a maneuver that can simulate fluid loading [66].

### Prediction of fluid responsiveness

Fluid administration is the first-line therapy in the early phases of shock states, except in patients with cardiogenic shock with pulmonary oedema [67, 68]. The main goal of fluid administration is to increase the systemic venous return pressure gradient, the cardiac preload, and ultimately cardiac output and oxygen delivery. Nevertheless, fluid administration increases cardiac output only in half of patients [69] and fluid accumulation is harmful in critically ill patients [70–73] and in patients with ARDS [74]. Therefore, is it currently recommended to assess fluid responsiveness in patients with shock after the initial phase of management [67, 68, 75]. Static markers of preload cannot reliably predict fluid responsiveness and dynamic tests have thus been developed to predict fluid responsiveness in patients under mechanical ventilation [76, 77], most of them being based on heart–lungs interactions [78]. The first dynamic test that has been developed is the respiratory variation of pulse pressure (PPV) [79], which is an easily obtained reflection of the respiratory variation of stroke volume, since for a constant arterial compliance, pulse pressure mainly depends on stroke volume [80]. If the right ventricle is preload-dependent, the decrease in its preload during mechanical insufflation should result in a decreased right ventricular stroke volume at the same time and thus in a decreased left ventricular preload during expiration due to the pulmonary transit time. This can in turn induce a decrease in left ventricular stroke volume if the left ventricle is preload-dependent. Therefore, the more the left ventricular stroke volume and the pulse pressure change during the mechanical ventilation cycle, the more likely the patient’s heart is preload-dependent and hence, the patient is fluid responsive [78, 79]. If one of the two ventricles is preload-independent, mechanical ventilation-induced changes in right ventricular preload do not result in significant changes in left ventricular stroke volume so that PPV is low. Many clinical studies confirmed the validity of these hypotheses in different clinical settings [81, 82], although several limitations exist in critically ill patients, the most frequent ones being low tidal volume ventilation, persistent spontaneous breathing activity, and cardiac arrhythmia [82–84]. It is noteworthy that in patients with ARDS, particularly when they are ventilated with high PEEP level, a high PPV might be related to right ventricular failure and to be a sign of right ventricular afterload dependence rather than fluid responsiveness. In this case of possible false positive PPV, it is suggested to assess the changes in PPV during a passive leg raising. If no change in PPV is observed, a high PPV indicates right ventricular afterload dependence, while a decrease in PPV suggests fluid responsiveness [82, 85].

Several other heart–lungs interaction tests have been developed to predict fluid responsiveness [77]. Some of them such as pulse contour-based stroke volume variation and respiratory variation of the inferior or superior vena cava diameters assessed by ultrasound imaging are not superior to PPV [86, 87] and share the same limitations as PPV [83, 84]. In patients ventilated with a tidal volume < 8 mL/kg, the tidal volume challenge can reliably predict fluid responsiveness by assessing the response of PPV to a brief increase in tidal volume (by 2 mL/kg) [88]. Recently, it was shown that the response of PPV to passive leg raising can also predict fluid responsiveness in cases of low tidal volume ventilation [89–91], even in the case of persistent spontaneous breathing activity [92]. This test and the tidal volume challenge have the advantage to require only an arterial catheter. Very recently, it has been shown the increase in cardiac output or the pulse pressure induced by a PEEP decrease may also reliably predict fluid responsiveness in patients with ARDS receiving low tidal volume ventilation [93].

## Conclusion

Heart–lungs interactions describe the interactions between the respiratory and the circulatory pump in the confined space of the thorax and result from the respiratory-induced changes in intrathoracic pressure, which are transmitted to the cardiac cavities and to the changes in alveolar pressure, which may impact the lung microvessels. Physiologically, heart–lungs interactions do not lead to significant hemodynamic consequences. In patients with acute respiratory failure, heart–lungs interactions may have significant hemodynamic consequences that can worsen the clinical conditions. The use of PEEP in patients mechanically ventilated for ARDS may result in hemodynamic compromise, especially when PEEP exerts excessive lung overdistension. The most recent application of heart–lungs interactions is the prediction of fluid responsiveness in mechanically ventilated patients. Numerous dynamic fluid responsiveness tests using heart–lungs interactions have been developed during the past years. They can help in the decision-making process regarding fluid administration and fluid removal, provided that their main limitations are well taken into consideration.

## Data Availability

Not applicable.

## Abbreviations

ARDS: Acute respiratory distress syndrome
COPD: Chronic obstructive pulmonary disease
FRC: Functional residual capacity
PEEP: Positive end-expiratory pressure
PPV: Respiratory variation of pulse pressure
WIPO: Weaning-induced pulmonary oedema
